# The relationship of childbirth experience with postpartum depression and anxiety: a cross-sectional study

**DOI:** 10.1186/s40359-023-01105-6

**Published:** 2023-03-03

**Authors:** Parivash Ahmadpour, Farnaz Faroughi, Mojgan Mirghafourvand

**Affiliations:** 1 Midwifery Department, Faculty of Nursing and Midwifery, Student Research Committee, Tabriz University of Medical Sciences, Tabriz, Iran; 2 Midwifery Department, Faculty of Nursing and Midwifery, Maragheh Branch, Islamic azad University, Maragheh, Iran; 3 Social Determinants of Health Research Center, Department of Midwifery, Faculty of Nursing & Midwifery, Tabriz University of Medical sciences, Tabriz, Iran; 4 Menopause Andropause Research Center, Ahvaz Jundishapur University of Medical Sciences, Ahvaz, Iran; 5 Social Determinants of Health Research Center, Tabriz University of Medical sciences, Tabriz, Iran

**Keywords:** Childbirth experience, Depression, Anxiety

## Abstract

**Background:**

The childbirth experience is a personal life event that is influenced by physiologic and mental-psychological processes. Due to the prevalence of psychiatric problems after childbirth, it is important to recognize the factors affecting women’s emotional reactions. This study was conducted to define the relationship of childbirth experience with postpartum anxiety and depression.

**Methods:**

This cross-sectional study was conducted on 399 women from 1 to 4 months after their childbirth who were referred to health centers in Tabriz-Iran from January 2021 to September 2021. Socio-demographic and obstetric characteristics questionnaire, Childbirth Experience Questionnaire (CEQ 2.0), Edinburgh Postpartum Depression Scale (EPDS), and Postpartum Specific Anxiety Scale (PSAS) were used to collect the data. The general linear modeling was used along with adjustment of socio-demographic characteristics to determine the relationship between the childbirth experience with depression and anxiety.

**Results:**

The mean (SD) of the overall score for childbirth experience, anxiety, and depression were 2.9 (0.2) (score range: 1 to 4), 91.6 (4.8) (score range: 0 to153), and 9.4 (0.7) (score range: 0 to 30), respectively. There was a significant inverse correlation between the overall score of childbirth experiences, the depression score (r= -0.36, p < 0.001), and the anxiety score (r= -0.12, p = 0.028) based on the Pearson correlation test. According to the general linear modeling and with adjustment of socio-demographic characteristics, with the increasing score of the childbirth experience, the depression score decreased (B= -0.2; 95%CI: -0.3 to -0.1). Moreover, the variable of control during pregnancy was a predictor for postpartum depression and anxiety, so in women with the control during pregnancy, the mean score of postpartum depression (B= -1.8; CI 95%: -3.0 to -0.5; P = 0.004) and anxiety (B=-6.0; CI 95%: -10.1 to -1.6; P = 0.007) was less.

**Conclusion:**

Based on the study results, postpartum depression and anxiety are related to childbirth experiences, therefore considering the effects of mothers’ mental health on other aspects of a woman and her family’s life, the core role of health care providers and policymakers in creating positive childbirth experiences is determined.

**Supplementary Information:**

The online version contains supplementary material available at 10.1186/s40359-023-01105-6.

## Background

Childbirth is an important and challenging life event, which affects many women annually. Birth events are stressful for most women and can include a wide spectrum of consequences and obstetric interventions [1]. After childbirth, women are vulnerable to postpartum psychiatric disorders, including depression, anxiety, and attachment disorders [2]. The experiences women achieve during the labor process are considered important childbirth consequences and these experiences will stay with them throughout their whole life [3].

Factors like the feeling of control and perceived control [4, 5], the extent of labor pain [6], caregiver’s support during the childbirth process [7], perinatal analgesia [8, 9], level of awareness, type of labor [10], and the amount of pregnant mother’s involvement in the decision-making process [6] influence women’s childbirth experiences. Moreover, unplanned medical interventions during the labor, such as emergency cesarean, assisted vaginal delivery [11, 12], mid-pregnancy problems [13], fear of childbirth [14], long-term labor [15], and the need for neonatal intensive care [16] are related to women’s dissatisfaction with childbirth. Different control and support aspects correlate with the childbirth experience, labor’s perception as a trauma, and depression [17, 18].

Physical changes during pregnancy contribute to a woman’s sense of pride in her own body and can be seen as very positive. However, these changes can also be perceived as threatening due to the uncontrollable process, which can lead to an increased sense of insecurity and fear related to childbirth. It has been shown that perceived insecurities during pregnancy and childbirth may adversely affect mothers’ ability to adjust to the responsibilities of parenthood, and their postpartum experiences [19, 20]. However, it is suggested that prenatal control influences on birth satisfaction by increasing healthy expectations and increasing the congruence between expectations and experienced control [21].

A new mother undergoes fundamental physiologic and emotional changes after labor and also faces the new responsibility of caring for the newly born infant [22]. Therefore, the first postpartum months are important periods in women’s lives, which can be associated with an important psychological disorder for women [23]. Depression and anxiety are common postpartum psychiatric disorders.

Postpartum depression is a general health problem and is seen in 10–15% of new mothers after childbirth [24]. Postpartum depression occurs as sleep disturbances, mood swings, changes in appetite, fear of hurting, serious concerns about the infant, too much sorrow and crying, feeling of doubt, difficulty in concentration, lack of interest in daily activities, death thoughts, and suicidal thoughts [25, 26]. Despair in the severe form of the disease can be life-threatening and result in suicide [27] and is responsible for 20% of the postpartum mortality of mothers [28]. Also, cases of fear of hurting the child (36%), weak attachment to the infant (34%), and even in severe cases, child suicide have been reported [29, 30]. Different risk factors have been proposed as postpartum depression risk factors, including marital conflicts [31], lack of self-confidence [32], difficult socio-psychological conditions [33], negative life events in a year before childbirth, and negative labor experiences [34]. Studies show that a negative childbirth experience will be associated with a request for a cesarean [35], depression during the next pregnancy [36], and postpartum depression [34].

Postpartum anxiety is one of the most common psychiatric problems that occur more often than postpartum depression and if left untreated, it can increase the risk of postpartum depression. The prevalence of anxiety disorder is between 9.9 and 20% in the first year after childbirth [37]. Anxiety can affect maternal abilities by creating irrational fears and limiting the mother’s activities. Also, studies show that maternal anxiety can prevent optimal mother-child interactions, inhibit the child’s socio-cognitive development and affect the infant’s nutritional outcomes and behaviors [37–39].

With increasing of postpartum psychological disorders, it is crucial to identify the effective factors on women’s emotional reactions. It is imperative to gather more information about how to minimize postpartum psychological distress and how to maximize positive emotions among women in order to reduce the incidence of postpartum psychological disorders [40]. In studies examining the link between gynecological factors and pregnancy and childbirth problems and postpartum depression, contradictory findings have been reported [41, 42]. Families suffering from postpartum depression suffer serious health consequences [43]. In order to take appropriate therapeutic measures, it is essential to identify the underlying causes of postpartum psychiatric disorders and susceptible individuals. Prior to developing screening programs and designing evidence-based preventive programs, scientific documents about the disease’s etiology and underlying factors need to be collected [44]. As a result, this study was designed to determine whether childbirth experiences are associated with anxiety and depression.

## Methods

### Study design, participants, and setting

This cross-sectional study was performed on 339 women from 1 to 4 months after their childbirth who were referred to health centers in Tabriz-Iran from January 2021 to September 2021.

Inclusion criteria included: having at least 18 years of age, residence in Tabriz, and women in 1 to 4 months after delivery. Women with the following criteria were not included in the study: multiple pregnancies, preterm labor (less than 37 weeks of pregnancy), and having a history of depression or psychological health problems during pregnancy or before it based on the mother’s statements.

In order to estimate the sample size in the present research, the formula for the estimation of a mean in a single population was employed. In the present study, the value of z with a 95% confidence level was 1.96, and the values of mean (SD) (mean score of childbirth experience) were 2.71 (0.73) based on the study of Ghanbari-Homayi et al. [45] and considering d (precision) equal to 0.05 around the mean, a sample size of 226 people was obtained. Due to the clustered nature of the sampling and regarding the design effect of 1.5, the final sample size was calculated to be 339.

### Sampling

Sampling was conducted after approving of this study by ethics committee of Tabriz University of Medical Sciences (Ethical code: IR.TBZMED.REC.1399.1109). Postpartum women referred to Tabriz health centers were the target population. There are 84 health centers in Tabriz. Cluster random sampling was used for sampling. We first randomly selected a quarter of the health centers (n = 21) using www.random.org. Birth-giving women were distributed differently across health centers. Probability proportional to size sampling was used in the sampling process. Based on the number of women covered by each center, the researcher selected a sample of women 1 to 4 months after birth. The number of selected samples from each center was determined by the number of women covered by each center, and then they were randomly selected. In Appendix 1, you can find a table that shows the number of clusters, the number of eligible mothers in each cluster, and the number of chosen participants in each cluster.

After selecting eligible individuals, the researcher contacted them by phone. If they wished, they were invited to attend the health center on a specific day. Following the presentation, the study’s objectives and methods were explained, and written informed consent was obtained if they wished to participate. We assured participants that their names, information, and results would remain confidential. The participants were interviewed face-to-face during this face-to-face session to complete socio-demographic and obstetric characteristics questionnaires, Childbirth Experience Questionnaires (CEQ2.0), Edinburgh Postpartum Depression Scales (EPDS), and Postpartum Specific Anxiety Scales in an Iranian population (PSAS-IR) in a relatively quiet environment.

### Data collection tools

The data of this research were collected by socio-demographic and obstetrics characteristics questionnaire, CEQ 2.0, EPDS, and PSAS-IR.

Socio-demographic and obstetrics characteristics questionnaire.

This questionnaire included questions about age, spouse’ age, the sufficiency of monthly income, living expenses, education of the woman and her spouse, occupation of the woman and her spouse, marital status, residence status, the mother’s weight, and height. The obstetrics profile included questions about the number of pregnancies, the number of deliveries, the number of abortions, the history of infertility, attending or not attending pregnancy classes, controlling over pregnancy and pregnancy status in terms of wanted or not, type of childbirth, and neonate’s admission at neonatal intensive care unit (NICU). Content and face validity were used to determine the validity of the questionnaire. The questionnaire was provided to the faculty members of the Schools of Nursing & Midwifery, and Medicine, and after collecting their comments, the instruments were modified based on the received feedback.

### Childbirth experience questionnaire 2.0 (CEQ 2.0)

The first version of this tool contained 23 statements, but the revised questionnaire by the inventor contains 25 statements, including 3 extra items. This tool measures the childbirth experience of women. The questionnaire includes the following areas: personal capacity (sense of control, personal feelings about childbirth and labor pain), professional support (information and midwifery care), perceived safety (feeling of security and memories of childbirth), and participation (the woman’s ability to change posture, movements, and pain relief during labor and delivery). Of 25 items in the questionnaire, 23 statements are completed as multiple-choice questions and 3 statements are completed in visual analog scale (VAS). The validity and reliability of this tool have been proven in the American female population. The answers are as strongly agree (score 1), often agree (score 2), often disagree (score 3), and strongly disagree (score 4). Questions answered on a visual scale will be converted to a score of 1 to 4: scores 0–40 (score 1), scores 41–60 (score 2), 61–80 (score 3), and 81–100 (Score 4). Sentences with negative concepts (experiencing severe pain, feeling tired, scared, and having bad memories) are scored negatively. The high mean scores in this tool mean a more positive experience of childbirth [46]. The validity and reliability of the Persian version of this questionnaire in the research environment of this study have been determined by Ghanbari-Homayi et al. and its Cronbach’s alpha was 0.93 and the intra-correlation coefficient (ICC) was 0.97 [47].

Edinburgh Postpartum Depression Scale (EPDS).

The questionnaire is used to measure pregnancy and postpartum depression and was developed by Cox et al. in 1987. This tool consists of 10 multiple-choice questions, in which the choices have been organized from low to high (1, 2, 4) and in some from high to low (3, 5, 6, 7, 8, 9, 10). The options for each question are assigned a score from zero to three based on the intensity of the symptom and the score a person obtained from a total of ten questions can vary from zero to 30. Mothers who score above the 12 thresholds have varying degrees of depression. The validity of this scale was calculated to be equal to 0.78 using the simultaneous correlation coefficient of this scale and the Beck depression scale and the reliability of this scale was estimated equal to 0.75 by Cronbach’s alpha method and Bisection method [48]. Montazeri et al. also reported a Cronbach’s alpha of 0.77 for this questionnaire during pregnancy [49].

Postpartum Specific Anxiety Scale (PSAS).

This questionnaire designed by Fallon et al., Consists of 51 3-point Likert questions, including anxieties related to the mother’s competence and attachment, anxieties related to the infant’s safety and welfare, and anxieties about the practical care of the infant [50]. The validity and reliability of the Persian version of this questionnaire have been confirmed by Mirghafourvand et al. and Cronbach’s alpha coefficient and ICC have been reported to be 0.93 and 0.92, respectively [51].

### Data analysis

In order to analyze the data, SPSS statistical software (version 25) was used. Socio-demographic, obstetric, anxiety, depression, and childbirth experiences of the participants were described through descriptive statistics such as frequency, percentage, mean, and standard deviation. In order to determine whether quantitative data were normal, we used both visual inspection and Skewness and Kurtosis, which had a normal distribution. A Pearson correlation test was carried out to assess the relationship of anxiety and depression with childbirth experience. Independent t-test and one-way analysis of variance were performed to examine the relationship between socio-demographic and obstetrical characteristics and anxiety and depression variables. We then used general linear modeling to estimate the effect of the independent variables (socio-demographic and obstetrical characteristics as potential confounders, and childbirth experience) on the dependent variable (anxiety and depression). The cluster effect was controlled using R software on the dependent variables (anxiety and depression).

## Results

A number of 521 women were studied. Of these, 399 women were eligible and 339 were included in the study with an 85% participation rate. The mean (SD) age of participants was 28.7 (6.0) years. Less than half of the women (45.7%) had secondary education and the majority (82.3%) were housewives. The mean (SD) age of the spouses was 33.9 (6.6) years and the majority of them (78.8%) had primary and secondary education. Moreover, about half of the spouses (54.6%) were self-employed. About half of the women (47.2%) reported that their monthly income was relatively sufficient for living expenses. More than half of the participants (60.5%) lived in their private homes. About one-third of women (35.7%) experienced their first pregnancy and the method of delivery was vaginal delivery in about half of the women (49.0%) (Table 1).

**Table 1:** Socio-demographic characteristics of the participants (n = 339)

characteristics of Socio-demographic		Mean (SD)	Relationship with Depression		Relationship with Anxiety	
			R	P-Value	r	P-Value
**Age** (Year)		28.7 (6.0)	0.12	0.000	-0.04	0.418
**Spouse age** (Year)		33.9 (6.6)	0.11	0.047	-0.05	0.349
**Married age** (Year)		20.9 (5.4)	0.12	0.023	-0.07	0.190
**Body mass index** (kg/m^2^)		25.6 (4.8)	-0.06	0.238	-0.04	0.470
**Gestational age** (Week)		38.6 (1.0)	0.02	0.755	0.03	0.617
		**Number (Percent)**	**Mean (SD)**	**P-Value**	**Mean (SD)**	**P-Value**
**Sufficiency of income for living expenses**				0.001		0.903
Completely sufficient		101 (29.8)	9.1 (5.7)		92.3 (20.5)	
Somewhat sufficient		160 (47.2)	8.6 (5.9)		91.1 (19.2)	
Insufficient		78 (23.0)	11.6 (6.5)		91.7 (20.8)	
**Home status**				0.117		0.062
Private house		205 (60.5)	9.0 (5.6)		91.3 (19.5)	
Corporate home		112 (23.0)	9.0 (6.4)		90.3 (18.4)	
Living in a relatives’ house		22 (6.5)	11.5 (8.1)		101.1 (28.3)	
**Education**				0.103		0.601
Primary school		131 (38.6)	8.4 (5.8)		92.0 (20.6)	
Secondary school		155 (45.7)	9.8 (6.4)		91.7 (20.1)	
High school		40 (11.8)	11.2 (5.8)		92.5 (19.2)	
Diploma		12 (3.5)	10.1 (5.0)		82.4 (13.6)	
University		1 (0.3)	11 (0.0)		97.0 (0.0)	
**Spouse education**				0.657		0.119
Primary school		135 (39.8)	9.3 (6.1)		93.2 (20.6)	
Secondary school		132 (38.9)	9.9 (6.3)		91.7 (19.4)	
High school		55 (16.2)	8.9 (5.8)		86.1 (19.4)	
Diploma		17 (5.0)	8.7 (4.9)		96.1 (18.3)	
**Job**				0.003		0.820
Housewife		279 (82.3)	8.9 (5.9)		91.6 (19.8)	
Working at home		57 (16.8)	12.1 (6.6)		90.9 (21.0)	
Working abroad		3 (0.9)	23.0 (2.8)		99.0 (2.8)	
**Spouse employment**				0.488		0.159
Unemployed		12 (3.5)	8.3 (5.9)		82.3 (22.1)	
Employee		49 (14.5)	8.5 (4.9)		90.2 (20.5)	
Manual worker		93 (27.4)	9.3 (6.1)		89.9 (18.5)	
Self-employment		185 (54.6)	9.9 (6.4)		93.5 (20.3)	
**Gravid**				0.172		0.296
1		121 (35.7)	8.6 (5.9)		89.3 (18.7)	
2		118 (34.8)	9.9 (6.3)		92.8 (19.8)	
≥ 3		100 (29.5)	9.8 (6.0)		92.9 (21.4)	
**Abortion**				0.005		0.425
Yes		236 (69.6)	8.8 (5.7)		91.0 (19.2)	
No		103 (30.4)	10.8 (6.6)		92.9 (21.6)	
**Control during pregnancy**				0.001		0.004
No		175 (51.6)	10.8 (6.3)		89.8 (4.4)	
Yes		164 (48.4)	8.1 (5.5)		86.2 (4.0)	
**Prenatal education**				0.476		0.107
No		305 (90.0)	9.5 (6.1)		86.4 (20.8)	
Yes		34 (10.0)	8.7 (6.3)		92.2 (19.8)	
**Mode of Delivery**				0.247		0.247
Normal Vaginal delivery (NVD)		166 (49.0)	9.0 (6.2)		90.8 (20.7)	
Elective Cesarean Section		118 (34.8)	10.1 (5.6)		93.9 (17.9)	
Emergency Cesarean Section		55 (16.2)	9.8 (6.7)		89.5 (21.7)	
**Neonatal NICU admission**				0.252		0.260
No		283 (83.5)	9.6 (6.3)		94.4 (18.2)	
Yes		56 (16.5)	5.6 (5.0)		91.1 (20.3)	

The mean (SD) of the total scores for childbirth experience, anxiety, and depression were 2.9 (0.2) (score range: 1 to 4), 91.6 (4.8) (score range: 0 to 30), and 9.4 (0.7) (score range: 0 to 153), respectively. According to the Pearson correlation test, there was a significant inverse correlation between the total score of childbirth experience and the score of depression (r= -0.36, P < 0.001) and anxiety (r = -0.12, P = 0.028) (Table 2). Less than a third of the women (28.0%) had depression with a score higher than 12.

**Table 2:** Childbirth experience, depression and anxiety status and the relationships of childbirth experience with depression and anxiety (n = 339)

Variable	Mean (SD) ^*^	Obtained score range	Obtainable score range	Relationship with Childbirth experience	
				**r**	**P** ^§^
Anxiety	91.6 (4.8)	60 to 150	0 to 153	-0.12	0.028
Depression	9.4 (0.7)	0 to 27	0 to 30	-0.36	˂0.001
Childbirth experience	2.9 (0.2)	1.64 to 3.68	1 to 4	-	-

^*^ Mean (Standard Deviation (

Based on the results of bivariate tests (one-way ANOVA and independent t-test), there was a statistically significant relationship between the depression score with the variables of age (P = 0.001), age of the spouse (P = 0.047), age of marriage (P = 0.023), sufficiency of monthly income, for living expenses (P = 0.001), occupation (P = 0.003), abortion (P = 0.005) and control during pregnancy (P = 0.001). Also, there was a statistically significant relationship between the anxiety score with the variables of spouse education (P = 0.038) and control during pregnancy (P = 0.004). These variables were included in the general linear modeling together with the variable of childbirth experiences as an independent variable and the results of the adjusted general linear modeling showed that the variables of control during pregnancy, childbirth experience, and age had a statistically significant relationship with the depression (P < 0.05) so that with increasing the score of the childbirth experience, the mean score of depression decreased (B=-0.2; 95%CI:-0.278 to -0.143). In addition, postpartum depression was lower in women with control during pregnancy (B=-1.8; CI 95%: -3.0 to -0.5; P = 0.004) as well as the depression score increased with age too (B = 0.05, CI 95%: 0.2 to 0.4 p = 0.013). Besides, the variable of control during pregnancy had a statistically significant relationship with anxiety (P < 0.05), as the postpartum anxiety was less in women with control during pregnancy (B=-6.0; 95%CI: -10.1 to -1.6; P = 0.007) (Tables 3 and 4).

**Table 3:** Predictors of depression based on general linear modeling (n = 339)

Variable		Childbirth experience		
		B (95% CI*)	P	
**Childbirth experience**		-0.2 (-0.3 to -0.1)	< 0.001	
**Age** (Year)		0.2 (0.05 to 0.4)	0.013	
**Spouse Age** (Year)		-0.4 (-0.2 to 0.1)	0.552	
**Married Age** (Year)		-0.1 (-0.2 to 0.05)	0.175	
**Control during pregnancy** (Reference: No)				
Yes	-1.8 (-3.0 to -0.6)			0.004
**Sufficiency of income for living expenses** (Reference: Completely sufficient)				
Insufficient	-1.2 (-3.0 to 0.5)			0.162
Somewhat sufficient	-1.1 (-2.8 to 0.5)			0.187
**Job** (Reference: Working abroad**)**				
Housewife	3.2 (-3.2 to 9.5)			0.325
Working at home	5.0 (-1.4 to 11.4)			0.125
**Abortion** (Reference: Yes)				
No	-1.12 (-2.4 to 0.2)			0.093

* 95% Confidence Interval

**Table 4:** Predictors of anxiety based on general linear modeling (n = 339)

Variable		Childbirth experience		
		B (95% CI*)	P	
**Control during pregnancy** (Reference: No)				
Yes	-6.0 (-10.1 to -1.6)			0.007
**Spouse education** (Reference: Diploma)				
Primary school	-1.2 (-11.3 to 8.8)			0.811
Secondary school	-2.5 (-12.5 to 7.6)			0.631
High school	-8.6 (-19.4 to 2.2)			0.120

* 95% Confidence Interval

## Discussion

The results of the present study showed that there was a significant inverse correlation between the overall score of the childbirth experience with depression, and anxiety so with increasing the score of the childbirth experience, the score of postpartum depression and anxiety decreases. Moreover, the variables of the childbirth experience, control during pregnancy, and age were predictors of postpartum depression and the control during pregnancy variable was a predictor of maternal anxiety.

The prevalence of depression in the present study was estimated to be 28.0%. In a systematic review in 2013, the pooled prevalence of PPD in Iran was 25.3% [52]. In another systematic review, the prevalence of depression was 17% with the highest prevalence (26%) in the Middle-East and the lowest prevalence (8%) in the Europe [53]. Iran is located in the Middle-East region and the reported prevalence in both mentioned systematic reviews is almost similar to our study.

The childbirth experience was one of the predictors of depression in the present study. In line with the results of the present study, a study conducted by Bell et al. in the United Kingdom to determine the relationship between depression and labor factors, the type of labor (physiologic delivery without intervention against difficult labor], and childbirth experiences. The results of the study showed that the symptoms of postpartum depression were related to the type of delivery, immediate postpartum complications, and the mother’s perception of the recent childbirth experience. Also, a more negative perception of the recent childbirth experience was associated with severe anxiety symptoms [54]. In a study conducted by Astbury et al. [55] which was aimed at determining the possible causes of postpartum depression, the results showed that negative labor experience and lack of participation in decision-making during labor were significantly associated with depression.

In the present study, depression was less in women who had control during pregnancy. Control issues during pregnancy and childbirth show themselves in three ways, including prenatal monitoring of fetal health during pregnancy, delivery and childbirth control, and the actual experienced control during delivery [56]. Pregnant women who have high control during pregnancy are more likely to attend childbirth classes and reduce their smoking, caffeine, and drug abuse. High internal control in pregnancy may help in better childbirth outcomes and higher satisfaction from the labor [57]. Research shows that high experiences of control during labor and birth increase birth satisfaction and reduce the incidence of postpartum depression. Lack of control is associated with unpleasant experiences of childbirth, postpartum depression, and post-traumatic stress disorder (PTSD) symptoms [17, 38].

A lower level of anxiety was observed in women who had control over their pregnancy in the present study. Studies have shown that anxiety disorders are more prevalent than depression in the postpartum period, with 16% of women suffering from anxiety disorders such as panic disorder, phobia, acute adaptation disorder, or postpartum depression. There is also a tendency for anxiety disorders to be associated with depression [58]. It is essential to control physical and emotional reactions to stress according to psychiatric theories of stress responses, depression, and PTSD. As a result of low levels of control [both internal and external] during an extremely stressful health event, depression can result, and an uncertain sense of control can lead to anxiety [59].

The information obtained from this study can be effective in improving the quality of services provided to pregnant mothers. Given the impact of postpartum anxiety and depression on family and community health, it is expected that by identifying the underlying causes of these health problems, an important step can be taken to improve maternal health. With the support and control of mothers in childbirth, we may be able to create more positive labor experiences for them [44]. The results of the present study provide an opportunity to look specifically at the impact of childbirth experiences that may be important for clinical interventions and policymaking.

One of the limitations of this study is due to its cross-sectional nature, as the relationships shown between the childbirth experience, depression, and anxiety, and some socio-demographic characteristics do not accurately indicate a causal relationship. In addition, the present study was conducted under COVID-19 pandemic conditions. These conditions negatively affect the psyche of humans, notably mothers in the postpartum period [60]. This study has the advantage of using standard tools. There is another strength to this study, which is its random sampling, which makes the study findings generalizable. However, the multicultural context of Iran will influence the human psyche [61], so similar studies need to be conducted in other provinces.

## Conclusion

According to the results of the study, childbirth experience and control during pregnancy are predictive factors of postpartum depression, and control during pregnancy is a predictive factor of postpartum anxiety. Due to the impact of the mother’s mental health on other aspects of a woman and her family’s life, by better caring for mothers during childbirth and creating positive childbirth experiences, women can be better prepared to face the challenges of the postpartum period, and this identifies the core role of midwives, midwifery trainers, and health care policymakers in helping to create positive childbirth experiences according to the mothers’ psychological needs.

## Electronic supplementary material

Below is the link to the electronic supplementary material.

Supplementary Material 1

## Data Availability

The datasets generated and/or analysed during the current study are not publicly available due to limitations of ethical approval involving the patient data and anonymity but are available from the corresponding author on reasonable request.

## Abbreviations

CEQ 2.0: Childbirth Experience Questionnaire
EPDS: Postpartum Depression Scale
PSAS-IR: Anxiety Scale in an Iranian population
NICU: neonatal intensive care unit
VAS: visual analogue scale
ICC: intra-correlation coefficient
SD: standard definition
ANOVA: Analysis of variance
COVID-19: coronavirus disease of 2019

## References

[1] Wilson RE, Salihu HM (2007). The paradox of obstetric” near misses”: converting maternal mortality into morbidity. Int J Fertil Womens Med.

[2] Brockington I (2004). Postpartum psychiatric disorders. The Lancet.

[3] Waldenström U (2003). Women’s memory of childbirth at two months and one year after the birth. Birth.

[4] Green JM, Baston HA (2003). Feeling in control during labor: concepts, correlates, and consequences. Birth.

[5] Ford E, Ayers S, Wright DB (2009). Measurement of maternal perceptions of support and control in birth (SCIB). J Womens Health.

[6] Hodnett ED (2002). Pain and women’s satisfaction with the experience of childbirth: a systematic review. Am J Obstet Gynecol.

[7] Hodnett ED. Caregiver support for women during childbirth.Cochrane Database Syst Rev. 2000:CD000199-CD. 10.1002/14651858.CD000199 11869571

[8] Waldenstrom U, Hildingsson I, Rubertsson C, Radestad I (2004). A negative birth experience: prevalence and risk factors in a national sample. Birth.

[9] Hidaka R, Callister LC (2012). Giving birth with epidural analgesia: the experience of first-time mothers. J Perinat Educ.

[10] Bryanton J, Gagnon AJ, Johnston C, Hatem M (2008). Predictors of women’s perceptions of the childbirth experience. JOGN Nurs.

[11] Andersen LB, Melvaer LB, Videbech P, Lamont RF, Joergensen JS (2012). Risk factors for developing post-traumatic stress disorder following childbirth: a systematic review. Acta Obstet Gynecol Scand.

[12] Wiklund I, Edman G, Ryding EL, Andolf E (2008). Expectation and experiences of childbirth in primiparae with caesarean section. Int J Gynaecol Obstet.

[13] Berg M, Lundgren I, Lindmark G (2003). Childbirth experience in women at high risk: is it improved by use of a birth plan?. J Perinat Educ.

[14] Henriksen L, Grimsrud E, Schei B, Lukasse M (2017). Factors related to a negative birth experience - A mixed methods study. Midwifery.

[15] Nystedt A, Högberg U, Lundman B (2005). The negative birth experience of prolonged labour: a case-referent study. J Clin Nurs.

[16] Smarandache A, Kim TH, Bohr Y, Tamim H (2016). Predictors of a negative labour and birth experience based on a national survey of canadian women. BMC Pregnancy Childbirth.

[17] Ford E, Ayers S, Wright DB (2009). Measurement of maternal perceptions of support and control in birth (SCIB). J Womens Health.

[18] Bryanton J, Gagnon AJ, Johnston C, Hatem M (2008). Predictors of women’s perceptions of the childbirth experience. J Obstet Gynecol Neonatal Nurs.

[19] Bronte-Tinkew J, Ryan S, Carrano J, Moore KA (2007). Resident fathers’ pregnancy intentions, prenatal behaviors, and links to involvement with infants. J Marriage Fam.

[20] Kuljanić K, Dorčić TM, Bistrović IL, Brnčić-Fischer A (2016). Prospective fathers: psychosocial adaptation and involvement in the last trimester of pregnancy. Psychiatr Danub.

[21] Goodman P, Mackey MC, Tavakoli AS (2004). Factors related to childbirth satisfaction. J Adv Nurs.

[22] O’Hara MW, Zekoski EM, Philipps LH, Wright EJ (1990). Controlled prospective study of postpartum mood disorders: comparison of childbearing and nonchildbearing women. J Abnorm Psychol.

[23] Mowbray CT, Oyserman D, Zemencuk JK, Ross SR (1995). Motherhood for women with serious mental illness: pregnancy, childbirth, and the postpartum period. Am J Orthopsychiatry.

[24] Kendell R (1985). Emotional and physical factors in the genesis of puerperal mental disorders. J Psychosom Res.

[25] Aswathi A, Rajendiren S, Nimesh A, Philip RR, Kattimani S, Jayalakshmi D (2015). High serum testosterone levels during postpartum period are associated with postpartum depression. Asian J Psychiatr.

[26] Norhayati M, Hazlina NN, Asrenee A, Emilin WW (2015). Magnitude and risk factors for postpartum symptoms: a literature reviewJ. Affect Disord.

[27] Youn J-H, Jeong IS (2011). Predictive validity of the postpartum depression predictors inventory-revised. Asian Nurs Res.

[28] Chaudron LH (2003). Postpartum Depression. Pediatr Rev.

[29] Thorsteinsson EB, Loi NM, Moulynox AL (2014). Mental health literacy of depression and postnatal depression: a community sample. Open J Depress.

[30] Spinelli MG (2004). Maternal infanticide associated with mental illness: prevention and the promise of saved lives. Am J Psychiatry.

[31] Gotlib IH, Whiffen VE, Wallace PM, Mount JH (1991). Prospective investigation of postpartum depression: factors involved in onset and recovery. J Abnorm Psychol.

[32] Zekoski EM, O’Hara MW, Wills KE (1987). The effects of maternal mood on mother-infant interaction. J Abnorm Child Psychol.

[33] Romans SE, Walton V, McNoe B, Herbison G, Mullen P (1993). Otago Women’s Health Survey 30-Month Follow-Up: I: onset patterns of non-psychotic Psychiatric disorder. Br J Psychiatry.

[34] Righetti-Veltema M, Conne-Perréard E, Bousquet A, Manzano J (1998). Risk factors and predictive signs of postpartum depression. J Affect Disord.

[35] Hildingsson I, Rådestad I, Rubertsson C, Waldenström U (2002). Few women wish to be delivered by caesarean section. BJOG.

[36] Rubertsson C, Waldenström U, Wickberg B (2003). Depressive mood in early pregnancy: prevalence and women at risk in a national swedish sample. J Reprod Infant Psychol.

[37] Dennis C-L, Falah-Hassani K, Shiri R (2017). Prevalence of antenatal and postnatal anxiety: systematic review and meta-analysis. Br J Psychiatry.

[38] McBean AL, Montgomery-Downs HE (2015). Diurnal fatigue patterns, sleep timing, and mental health outcomes among healthy postpartum women. Biol Res Nurs.

[39] Stein A, Pearson RM, Goodman SH, Rapa E, Rahman A, McCallum M (2014). Effects of perinatal mental disorders on the fetus and child. Lancet.

[40] Ford E, Ayers S (2009). Stressful events and support during birth: the effect on anxiety, mood and perceived control. J Anxiety Disord.

[41] Martin ME (1977). A maternity hospital study of psychiatric illness associated with childbirth. Ir J Med Sci.

[42] O’neill T, Murphy P, Greene V (1990). Postnatal depression–aetiological factors. Ir J Med Sci.

[43] Mathisen SE, Glavin K, Lien L, Lagerløv P (2013). Prevalence and risk factors for postpartum depressive symptoms in Argentina: a cross-sectional study. Int J Womens Health.

[44] De Crescenzo F, Perelli F, Armando M, Vicari S (2014). Selective serotonin reuptake inhibitors (SSRIs) for post-partum depression (PPD): a systematic review of randomized clinical trials. J Affect Disord.

[45] Ghanbari-Homayi S, Fardiazar Z, Meedya S, Mohammad-Alizadeh-Charandabi S, Asghari-Jafarabadi M, Mohammadi E (2019). Predictors of traumatic birth experience among a group of iranian primipara women: a cross sectional study. BMC Pregnancy Childbirth.

[46] Dencker A, Taft C, Bergqvist L, Lilja H, Berg M (2010). Childbirth experience questionnaire (CEQ): development and evaluation of a multidimensional instrument. BMC Pregnancy Childbirth.

[47] Ghanbari-Homayi S, Dencker A, Fardiazar Z, Jafarabadi MA, Mohammad-Alizadeh-Charandabi S, Meedya S (2019). Validation of the iranian version of the childbirth experience questionnaire 2.0. BMC Pregnancy Childbirth.

[48] Cox JL, Holden JM, Sagovsky R (1987). Detection of postnatal depression: development of the 10-item Edinburgh postnatal depression scale. Br J Psychiatry.

[49] Montazeri A, Torkan B, Omidvari S (2007). The Edinburgh postnatal depression scale (EPDS): translation and validation study of the iranian version. BMC Psychiatry.

[50] Fallon V, Halford JCG, Bennett KM, Harrold JA (2016). The postpartum specific anxiety scale: development and preliminary validation. Arch Womens Ment Health.

[51] Hasanzadeh R, Jafarabadi MA, Hasanpour S, Fallon V, Silverio SA, Montazeri R (2021). Psychometric evaluation of the postpartum specific anxiety scale in an iranian population (PSAS-IR). BMC Pregnancy Childbirth.

[52] Veisani Y, Delpisheh A, Sayehmiri K, Rezaeian S (2013). Trends of postpartum depression in Iran: a systematic review and meta-analysis. Depress Res Treat.

[53] Shorey S, Chee CYI, Ng ED, Chan YH, Tam WWS, Chong YS (2018). Prevalence and incidence of postpartum depression among healthy mothers: a systematic review and meta-analysis. J Psychiatr Res.

[54] Bell A, Carter C, Davis J, Golding J, Adejumo O, Pyra M (2016). Childbirth and symptoms of postpartum depression and anxiety: a prospective birth cohort study. Arch Womens Ment Health.

[55] Astbury J, Brown S, Lumley J, Small R (1994). Birth events, birth experiences and social differences in postnatal depression. Aust J Public Health.

[56] Weisman CS, Hillemeier MM, Chase GA, Misra DP, Chuang CH, Parrott R (2008). Women’s perceived control of their birth outcomes in the Central Pennsylvania Women’s Health Study: implications for the use of preconception care. Womens Health Issues.

[57] Schempf AH, Strobino DM (2009). Drug use and limited prenatal care: an examination of responsible barriers. Am J Obstet Gynecol.

[58] Matthey S, Barnett B, Howie P, Kavanagh DJ (2003). Diagnosing postpartum depression in mothers and fathers: whatever happened to anxiety?. J Affect Disord.

[59] Walker J. Control and the psychology of health. Buckingham. Open University Press; 2001.

[60] Durankuş F, Aksu E. Effects of the COVID-19 pandemic on anxiety and depressive symptoms in pregnant women: a preliminary study.J Matern Fetal Neonatal Med. 2020:1–7. 10.1080/14767058.2020.1763946 32419558

[61] Simkin P (1996). The experience of maternity in a woman’s life. J Obstet Gynecol Neonatal Nurs.

