# Supplementary material for: The relationship of childbirth experience with postpartum depression and anxiety: a cross-sectional study
Source: BMC Psychol. 2023 Mar 3;11:58. doi: 10.1186/s40359-023-01105-6 (PMC9983514; doi:10.1186/s40359-023-01105-6)
Supplement: Supplementary file 1 — Supplementary Material 1 [file 40359_2023_1105_MOESM1_ESM.docx]

| cluster | Number of selected participants of each cluster | Number of eligible mothers in each cluster |
| --- | --- | --- |
|  | 18 | 38 |
|  | 21 | 45 |
|  | 24 | 51 |
|  | 11 | 23 |
|  | 20 | 43 |
|  | 11 | 23 |
|  | 25 | 53 |
|  | 21 | 45 |
|  | 16 | 34 |
|  | 13 | 28 |
|  | 7 | 15 |
|  | 14 | 30 |
|  | 14 | 30 |
|  | 16 | 34 |
|  | 13 | 28 |
|  | 12 | 25 |
|  | 16 | 34 |
|  | 15 | 32 |
|  | 14 | 30 |
|  | 21 | 45 |
|  | 17 | 36 |

**Appendix 1: The** number of clusters, number of eligible mothers in each cluster and number of selected participants of each cluster
